# Comparative Analysis of Early Class III Malocclusion Treatments—A Systematic Review and Meta-Analysis

**DOI:** 10.3390/children12020177

**Published:** 2025-01-30

**Authors:** Andrei Otel, José María Montiel-Company, Álvaro Zubizarreta-Macho

**Affiliations:** 1 Faculty of Dentistry, Alfonso X El Sabio University, 28691 Madrid, Spain; arusu@myuax.com (A.O.); amacho@uax.es (Á.Z.-M.); 2 Department of Stomatology, Faculty of Medicine and Dentistry, University of Valencia, 46010 Valencia, Spain; 3 Department of Surgery, Faculty of Medicine and Dentistry, University of Salamanca, 37008 Salamanca, Spain

**Keywords:** skeletal class III, interceptive, orthodontics, malocclusions, orthopedics

## Abstract

Several therapeutic methods have been proposed for early class III malocclusion treatment; however, the existing literature does not provide evidence on the most recommended techniques or materials. The aim of the present systematic review and meta-analysis was to summarize the clinical evidence on the efficacy of each method. The PRISMA recommendations were followed. Four databases were searched for articles published up to February 2024. Controlled trials, randomized or non-randomized clinical trials, and prospective or retrospective studies with a control group and a minimum follow-up of 6 months were included. The risk of bias was assessed using the Cochrane collaboration tool. Inconsistency was assessed using the Q test, with a significance level of *p* < 0.05 and a forest plot. A total of 61 articles were identified, and eight were included in the meta-analysis, which examined three parameters: Sella-Nasion-A (SNA), Sella-Nasion-B (SNB), and A Point-Nasion-B (ANB) values. The meta-analysis was carried out using the random effects model and the inverse variance method. The effect size was determined as the difference between the means of the SNA, SNB, and ANB values in the initial and final intervention groups or between the intervention group and control group. Statistical significance was assessed using the z-test and was declared when *p*-value < 0.05. The heterogeneity of the meta-analysis was analyzed using the Q test and the I^2^ statistical index. Publication bias was analyzed using the trim-and-fill method to adjust the skewness of the funnel plot. The risk of bias in the selected studies was assessed using the Cochrane collaboration tool to analyze the methodological quality assessment of the clinical trials. There were statistically significant differences between traditional maxillary disjunction and traction and the different types of Alt-RAMEC protocols, with the latter allowing greater skeletal corrections.

## 1. Introduction

The demand for orthodontic treatments is increasing as the health and expectations of our population improve. Patients receive treatment to enhance their dentofacial appearance, correct the occlusal relationship, and eliminate malocclusions that could harm their health [1].

Mandibular prognathism has received the attention of dentists for more than 200 years. In 1778, John Hunter described class III malocclusion as a phenomenon where “the lower jaw projects too far forward, so that the lower-anterior teeth pass in front of maxilla ones when the mouth is closed” [2]. In addition, the prevalence of class III is highly variable depending on ethnic origin; it is low in the Caucasian type, 1.5–5%, and high in the Asian type, where it reaches up to 19% or even up to 26.7, according to other reviews [3,4,5].

Currently, orthodontists are aware that “true” class III malocclusions, which are caused by mandibular prognathism, represent far fewer cases than once thought, with values between 9.4 and 25% [6,7]. Most skeletal class III cases are due to maxillary retrusion, with a prevalence that accounts for 40% of cases [8,9]. However, other forms of class III malocclusion may exist, such as mixed class III with mandibular prognathism and maxillary retrognathism [10], class III due to bilateral hypercondylia syndrome [8], class III pseudo-prognathism [11], or limit class III cases [12], but they represent a limited percentage.

The etiology of class III is multifactorial, as it is both hereditary [13,14] and functional (craniocervical posture, oral breathing, and tongue position) [15,16,17], and it is also influenced by chewing habits [18] and endocrine factors [19].

The extraoral, intraoral, and cephalometric clinical examinations of each type of malocclusion have their own criteria and values. Therefore, the meticulous execution of these tests is essential for the correct diagnosis of this type of class III malocclusion [20,21].

Research on therapeutic methods for early class III malocclusion treatments is essential in orthodontics to prevent occlusal trauma and temporomandibular joint disorders due to occlusal overload.

The Graber method is still updated for the disjunction of class III malocclusion [22]. The most commonly used method today was invented by Delaire and involves the disjunction and traction of the maxillary bone. However, Le Petit’s mask, which is described by patients as more comfortable to wear and by orthodontists as more easily adjustable, is being used more often [23,24]. A new version, the Alt-RAMEC protocol, which was originally designed to treat children with cleft lip and palate, quickly gave very positive results and began to be used daily by orthodontists [25]. The Frankel, Andresen, and Balters activators are other widely used techniques in temporary dentition [26,27]. Miniplates with bone anchorage are the most innovative therapeutic option. The method described by De Clerck involves the implantation of titanium plates at the base of the zygomatic bone and others on the anterolateral surface of the mandible; the two bilateral plates are connected to each other using orthopedic force elastics (October, 2009) [27].

Briefly, there are many therapeutic approaches for the treatment of class III malocclusions; however, there is still no common consensus regarding the most efficient device or method that could be used for class III malocclusion treatments.

The objective of the work was to find the maximally effective method for the treatment of skeletal class III; through the measurement of Sella-Nasion-A (SNA), Sella-Nasion-B (SNB), and A Point-Nasion-B (ANB) values.

## 2. Materials and Methods

### 2.1. Study Design and Registration

A literature search was carried out following the PRISMA (Preferred Reporting Items for Systemic Reviews and Meta-Analyses; http://www.prisma-statement.org) guidelines (accessed on 17 July 2024) for systematic reviews and meta-analyses. The review also complied with the PRISMA 2020 Checklist (PRISMA Checklist) [28] and was registered in the Inplasy database (International platform of registered systematic review and Meta-Analysis) registration number: 10.37766/inplasy2024.2.0119.

### 2.2. Literature Search Process

The search strategy was based on the following question (PICO): for young patients with class III malocclusion (P), do all the therapeutic methods (I) have the same effectiveness (C), or are there expected differences in the effectiveness of the different therapeutic options (O)? An electronic search was carried out using the databases PubMed-Medline, Scopus, Web of Science, and Medline (22 February 2024). The search covered all of the internationally published literature up to February 2024 and included the following medical terms: “Skeletal class III”, “Interceptive”, “orthodontics”, “malocclusions”, and “orthopedics”. The Boolean operators applied were “OR” and “AND”. The search terms were structured as follows: (“skeletal class III”) AND (((interceptive) AND (malocclusions)) AND (orthodontics OR orthopedics)). One researcher (A.O.) carried out the database research. The titles and abstracts were selected by applying the inclusion and exclusion criteria. One researcher (A.Z.M.) extracted the data for the relevant variables.

The systematic review was carried out by A.O. and A.Z.M.; additionally, two researchers who were not involved in the selection process carried out the subsequent meta-analysis.

### 2.3. Inclusion and Exclusion Criteria

The inclusion criteria for this study were as follows: controlled trials; randomized or non-randomized clinical trials; prospective or retrospective studies with a control group; studies related to the early treatment of skeletal class III; studies with a sample of patients aged up to 12 years of age with mixed dentition requiring orthopedic treatment; and studies with pre- and post-treatment cephalometric data. No restrictions regarding the year or language of publication were applied.

The exclusion criteria for this study were as follows: studies not related to the early treatment of skeletal class III; interviews of authors; case studies; reviews and updates of knowledge; systematic studies and meta-analyses that did not have a comparable analysis; studies that included patients aged over 12 years; studies that included patients with class II skeletal profiles, syndrome patients, or patients with special needs; and studies with a follow-up of less than 6 months.

### 2.4. Data Extraction

The following data were extracted from each study by independent reviewers: author and year of publication, title, journal in which the article was published, sample size (*n*), follow-up time and measurement procedure.

The skeletal values were analyzed using lateral teleradiography and cephalometric tracings, which were taken from each study before treatment, at the end of treatment, and after treatment. These variations in degrees were re-compiled in an Excel sheet for statistical analysis.

### 2.5. Risk of Bias

The risk of bias in the selected studies was assessed using the Cochrane collaboration tool for the methodological quality assessment of clinical trials [29]. Specifically, it used the RoB2 for assessing randomized clinical trials and the ROBINS-1 for retrospective studies with a control group. This tool consists of seven items that evaluate sequence generation, allocation concealment, participant blinding, assessment blinding, incomplete data, free selective reporting, and other sources of bias (Table 1).

### 2.6. Data Synthesis and Statistical Analysis

The meta-analysis was carried out using the random effects model and inverse variance method. The effect size was determined as the difference between the means of the SNA, SNB, and ANB estimates in the initial and final intervention groups or those between the intervention group and control group. Statistical significance was assessed using the z-test at a *p*-value < 0.05. The heterogeneity of the meta-analysis was analyzed using the Q test and the I^2^ statistical index. The difference between subgroups was analyzed using the Q test. The meta-analyses were represented using forest plots. Publication bias was analyzed using the trim-and-fill method to adjust the skewness of the funnel plot.

## 3. Results

### 3.1. Results of the Search Process

**Figure 1:**
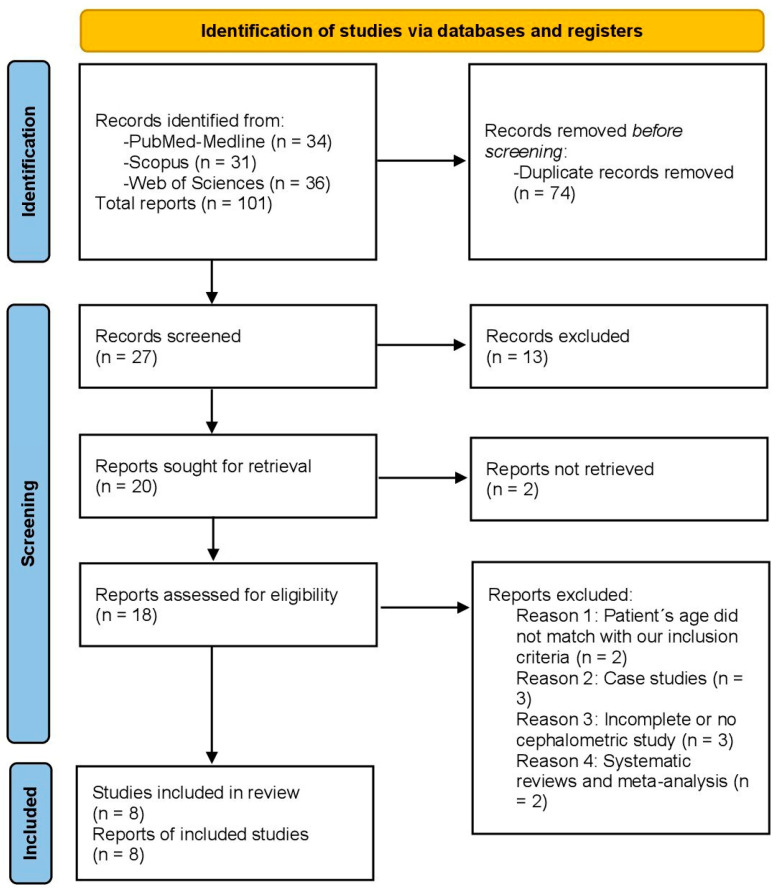
Preferred reporting items for systematic reviews and meta-analyses (PRISMA) flow diagram.

The systematic electronic search identified 34 articles in PubMed-Medline, 36 in Web of Science, and 31 in Scopus. Of the 101 articles, 74 were discarded as duplicates using RefWorks (https://refworks.proquest.com/reference/upload/recent/, accessed on 15 February 2024). After reading the titles and abstracts, an additional 13 articles were eliminated, leaving 20; 2 more articles were discarded because they were not sought for retrieval, leaving a total of 18 reports assessed for eligibility. A further 10 articles were rejected because they did not fulfill the inclusion criteria: the patient’s age did not match; they were simple case studies or were incomplete or had no cephalometric study; or, finally, they were systematic reviews and meta-analyses that were not compatible with our research. Finally, eight articles were included in the qualitative and quantitative synthesis because they included all the required data and variables (Figure 1).

### 3.2. Qualitative Analysis

The 11 included articles comprised randomized clinical trials, clinical trials, prospective studies, or retrospective studies. All of these studies were analyzed to determine the cephalometric changes in the SNA, SNB, and ANB values. Most of the studies included a sample size of approximately 20–40 patients, with subject ages ranging from 6 to 12 years old; some of the studies had follow-up times from 6 months up to 20 years (Table 2). Additionally, all of the teleradiographs of the selected studies were performed under similar conditions by practitioners using software cephalometric tracings, and no manual tracings were performed in the studies.

### 3.3. Quantitative Analysis

#### 3.3.1. SNA

**Figure 2:**
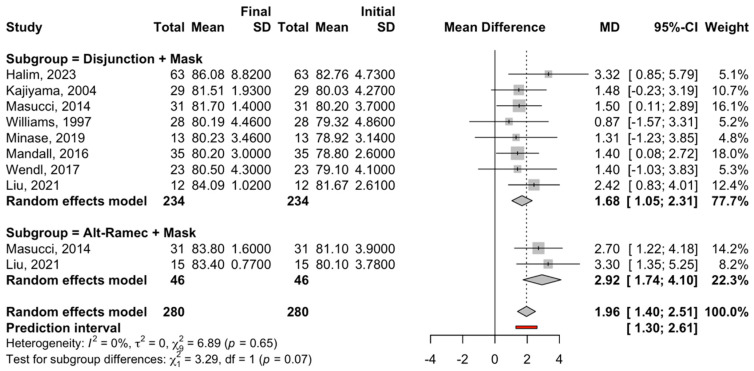
Forest plot of the meta-analysis of the mean difference in the SNA angle between the initial and final measurements for the treatment subgroups. SD, standard deviation: MD, median [30,31,32,33,34,35,36,37].

A subgroup meta-analysis was performed to estimate the effect size of the mean difference between the initial and final SNA values using a random effects model and the inverse variance method (Figure 2).

For the circuit breaker + face mask treatment subgroup, eight studies were combined to estimate a statistically significant increase of 1.68 degrees (95% confidence interval between 1.05 and 2.31) in the SNA angle (z-test = 5.2; *p*-value < 0.001) at the end of treatment. The meta-analysis of this subgroup did not show heterogeneity, with I^2^ = 0%, a Q test = 3.37, and a *p*-value = 0.848.

For the Alt-RAMEC + face mask treatment subgroup, two studies were combined to estimate a statistically significant increase of 2.92 degrees (95% confidence interval between 1.74 and 4.10) in the SNA value (z-test = 4.84; *p*-value < 0.001) at the end of treatment. The meta-analysis of this subgroup also did not show heterogeneity, with I^2^ = 0%, a Q test = 0.23, and a *p*-value = 0.631.

There were no significant differences between the two subgroups (Q test = 3.29; *p* = 0.065) (Figure 2).

Four RCT studies that compared the effect of disjunction + facial mask on SNA with a control group were combined to estimate a significant difference of 0.93 in favor of the intervention group, with a 95% confidence interval between 0.22 and 1.64 (z-test = 2.56; *p*-value < 0.011). The heterogeneity of the meta-analysis was high (I^2^ = 85.9% and Q test = 21.2; *p*-value < 0.0001) (Figure 2).

#### 3.3.2. SNB

**Figure 3:**
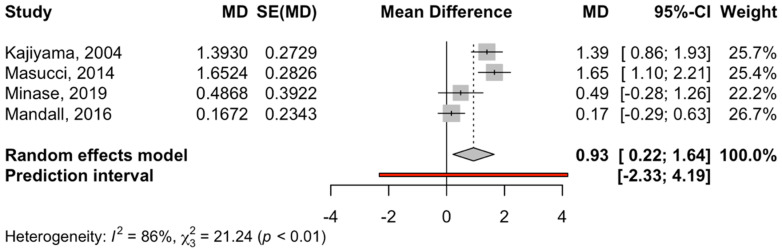
Forest plot of the mean difference in SNA between the disjunction and facial mask treatment group and the control group. MD, median; SE, standard error [31,32,34,36].

For the subgroup of disjunction treatment + facial mask, six studies were combined; the meta-analysis obtained a statistically significant decrease of −0.66 degrees (95% confidence interval between −1.32 and −0.01) in the SNB angle (z-test = −1.99; *p*-value = 0.047) at the end of treatment. The subgroup meta-analysis did not show heterogeneity, with I^2^ = 0%, a Q test = 2.38, and a *p*-value = 0.794 (Figure 3).

**Figure 4:**
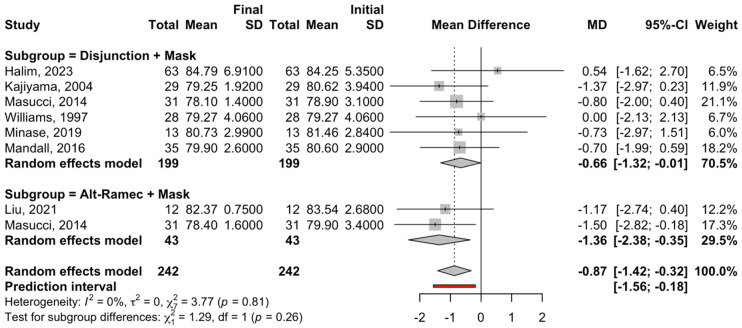
Forest plot of the meta-analysis of the mean difference in the SNB angle between the initial and final measurements for the treatment subgroups. SD, standard deviation: MD, median [30,31,32,33,34,35,36].

Regarding the Alt-RAMEC + face mask subgroup, two studies were combined to estimate a statistically significant decrease of −1.36 (95% confidence interval between −2.38 and −0.18), with a z-test = 4.84 and a *p*-value < 0.001 at the end of treatment. The meta-analysis of this subgroup did not show heterogeneity, with I^2^ = 0%, a Q test = 0.1, and a *p*-value = 0.753 (Figure 4).

There were no significant differences between the two subgroups (Q test = 1.29; *p* = 0.26).

**Figure 5:**
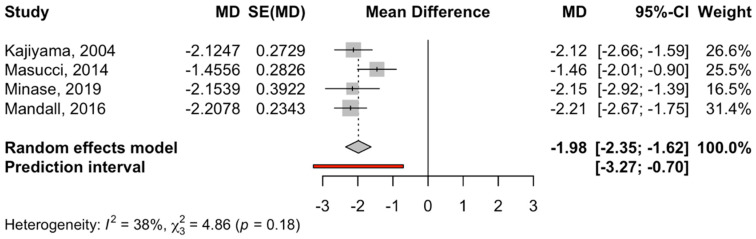
Forest plot of the mean difference in the SNB angle between the intervention group (disjunction and facial mask) and the control group. MD, median; SE, standard error [31,32,34,36].

Similarly, four RCT studies compared the effect of disjunction + facial mask on SNB with a control group [Figure 5, 95% confidence interval between −2.35 and −1.62 (z-test = −10.7; *p*-value = 0.001)]. The heterogeneity of the meta-analysis was minimal (I^2^ = 38.2% and Q test = 4.86; *p*-value = 0.183) (Figure 5).

#### 3.3.3. ANB

**Figure 6:**
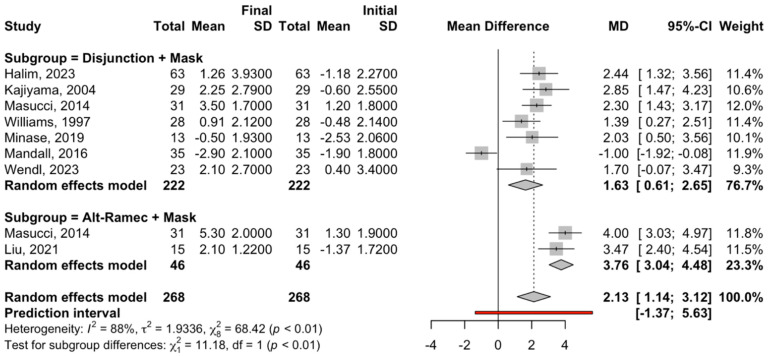
Forest plot of the meta-analysis of the mean difference in the ANB angle between the initial and final measurements for the treatment subgroups. SD, standard deviation: MD, median [30,31,32,33,34,35,36,37].

For the subgroup of disjunction treatment + facial mask, seven studies were combined; the meta-analysis obtained a statistically significant increase of 1.63 degrees (95% confidence interval between 0.61 and 2.65) in ANB (z-test = 3, 14; *p*-value = 0.002) at the end of treatment. Heterogeneity in the subgroup was high (I^2^ = 84.5% and Q test = 38.7; *p*-value < 0.001) (Figure 6).

Regarding the Alt-RAMEC + face mask subgroup, two studies were combined to estimate a statistically significant increase of 3.76 (95% confidence interval between 3.04 and 4.48), with a z-test = 10.26 and *p*-value < 0.001 at the end of treatment. The meta-analysis did not show heterogeneity, with I^2^ = 0%, a Q test = 0.52, and a *p*-value = 0.472 (Figure 6).

There were significant differences in ANB between the two subgroups (Q test = 11.1; *p* = <0.01).

**Figure 7:**
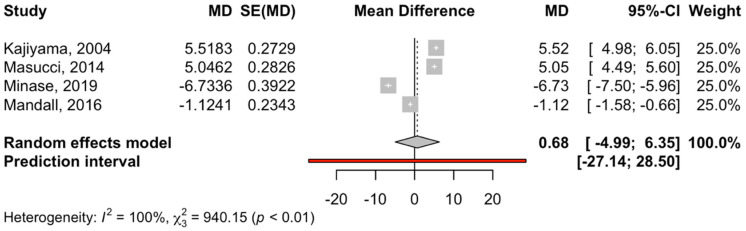
Forest plot of the difference in means of SNB between the intervention group (disjunction and facial mask) and control group. MD, median; SE, standard error [31,32,34,36].

The four RCT studies that compared the effect of disjunction + facial mask on ANB with a control group (Figure 7) showed values of −4.99 and 6.35 (z-test = −3.14; *p*-value = 0.001). The heterogeneity of the meta-analysis was high (I^2^ = 100% and Q test = 940.2; *p*-value < 0.001).

### 3.4. Publication Bias

#### 3.4.1. SNA

**Figure 8:**
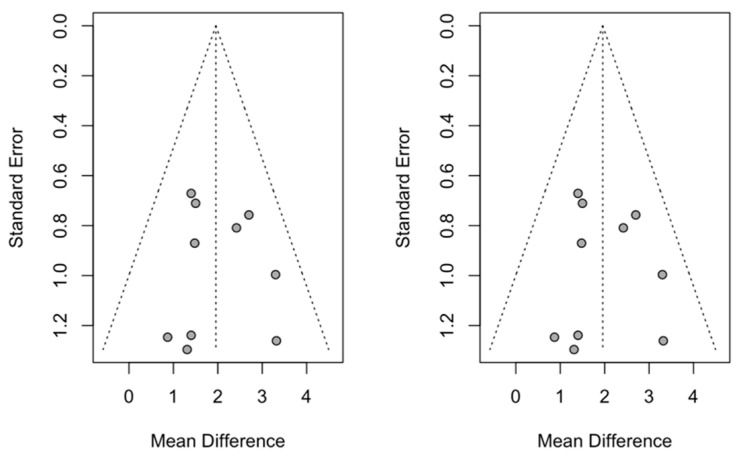
Funnel plot of the meta-analysis of the effect on SNA.

Publication bias was studied using the trim-and-fill method to adjust the skewness of the funnel plot; in this case (Figure 8), no studies were added; thus, the effect size was not changed in the meta-analyses of the effect on SNA.

#### 3.4.2. SNB

**Figure 9:**
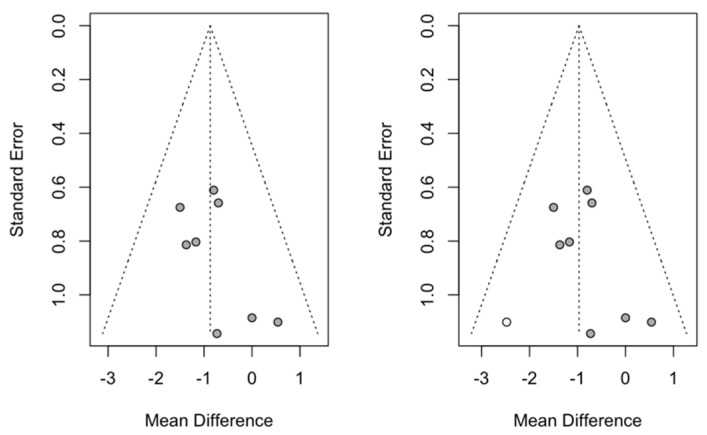
Funnel plot of the meta-analysis of the effect on SNB after adding one extra study (in white).

For the meta-analysis of the effect on SNB, a study was added to the funnel plot (Figure 9) that re-estimated the effect size and gave a statistically significant increase of −0.96 (with a 95% confidence interval between −1.50 and −0.45) with a z-test = 2.33 and a *p*-value = 0.019. The new estimate did not differ significantly from the previous one, which placed it at −0.87.

#### 3.4.3. ANB

**Figure 10:**
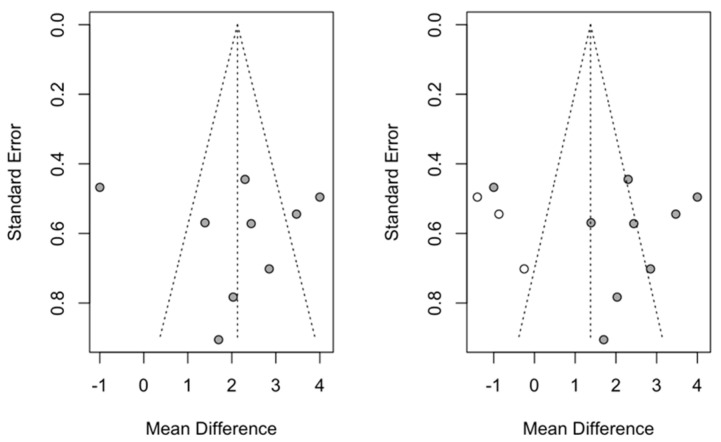
Funnel plot of the meta-analysis of the effect on SNB after the addition of three studies (shown as white circles).

For the meta-analysis of the effect on ANB, three studies were added to the funnel plot (Figure 10) that re-estimated the effect size and gave a statistically significant increase of 1.37 (with a 95% confidence interval between 0.31 and 2.40) with a z-test = 2.54 and a *p*-value < 0.011. This addition underestimated the effect size, although it did not differ significantly from the meta-analysis without the added studies, which placed it at 2.13.

## 4. Discussion

Skeletal class III malocclusions combined with maxillary retrognathism, mandibular prognathism, or both are often treated using an extraoral force application to correct the developing class III malocclusions. Currently, it is widely accepted that the early treatment of class III malocclusions is the most effective means of intercepting the incipient pathology [38,39,40].

Different therapeutic methods currently exist, ranging from 19th-century chin cups to the recent De Clerck mentoplates. At present, disjunction and maxillary traction with a facial mask are the most used methods worldwide [41,42,43]. To increase efficiency, an enhanced variant with an Alt-RAMEC protocol is also widely used [25]. These last two methods, as they are the most used, also have the most associated studies. Therefore, most of the data were extracted from disjunction/traction and Alt-RAMEC therapy studies.

Cephalometric values (SNA, SNB, and ANB) are the most objective and relevant parameters to consider for skeletal therapy improvements. Regarding SNA values (Figure 2), the Alt-RAMEC group reported more efficiency for point A displacement, with an average forward movement of 2.92° (95% confidence interval and *p*-value < 0.001). This is similar to the original result by Liou et al., who obtained a point A forward movement of 2.9° [25]. In 2010, Isci et al. obtained a movement of point A of 4.13° [44]. According to them, this protocol displaces the maxilla more anteriorly and disarticulates the circumaxillary sutures with more efficiency than the “conventional” expanders; it takes place later than maxillary traction and is twice as efficient. These results are in agreement with those of Zhou et al., who emphasize the increased efficiency of this method with regard to skeletal displacement compared with the conventional one [45].

Regarding the “conventional methods”, there was a point A forward movement of 1.68° with a 95% confidence interval. Among these results, the chin cup showed less efficiency than disjunction and expansion, with a point A movement of only 0.4°. However, the use of the chin cup is currently a somewhat controversial topic due to condyle harm. It was originally thought that the chin cup would produce a condyle growth inhibition, which could be a long-term side effect [46]. Indeed, mandibular growth restriction is supported by some authors [47,48,49] and contraindicated by others [50,51].

However, there is still a lot of controversy; many orthodontists have concluded that the chin cup has no effect on mandibular dimensions and is only associated with joint compression [52]. Barett et al. found only 50% efficacy in early mixed dentition, deducing that deciduous teeth might be a good starting point for this method [53]. In Wendl’s study, a 34% failure for chin cup use was obtained. For ease of interpretation, the data from chin cup use failures were transformed into control values, and successful treatments took the role of test values. More importantly, Abdelnaby [22] reported that the force of the chin cup was not statistically correlated with the skeletal displacement of points A and B.

The reverse twin block in Rohit’s study showed more efficiency than disjunction and traction, with a point A movement of 2°. As this is the only study to date that reports skeletal improvements, we cannot correlate or provide any interpretation of this method. Regarding the SNB angle (Figure 3, Figure 4 and Figure 5), both groups demonstrated statistically significant decreases. Regarding the Alt-RAMEC procedure, the reduction was more significant and was of the order of −1.36° at the end of treatment. The other procedures obtained a lower result, establishing a decrease of −0.66°. There were no significant differences between the two subgroups. It is important to emphasize that these values correspond with those of Liou et al., published in 2005; the author noted an SNB value of −2.2° value at the end of the maxillary traction period compared to only −0.9° with conventional disjunction and traction.

Regarding the ANB angle (Figure 6 and Figure 7), a statistically significant increase was noted in two subgroups (disjunction and traction and the Alt-RAMEC variant). First, concerning the Alt-RAMEC and traction variant, a significant increase of 3.76° was noted at the end of treatment. Then, in the subgroup of maxillary disjunction and traction, an increment of the ANB angle of 1.63° was noted at the end of treatment. Heterogeneity in the subgroup was high. The latter was due to the accumulation of differences in the SNA and SNB values and to the heterogeneity of the methods used, such as the chin cup, which had low efficiency.

The main limitation of the present study was the lack of full access to all articles relating to the topic of the early treatment of skeletal class III. Indeed, in the present study, we analyzed and interpreted the articles available in four databases (PubMed, Medline, Scopus, and Web of Science), which gave us access, thanks to institutional emails, to the full articles. The second limitation was due to the fact that only skeletal bone factors were considered when diagnosing the effectiveness of treatments. Indeed, to allow an effective analysis, we had to eliminate articles with incomplete cephalometric data and others with very interesting characteristics; these characteristics were specific to these analyses, and therefore, it was not possible to utilize them. They included, for example, dento-alveolar characteristics such as Incisor Mandibular Plane Angle (IMPA) (Minase, Himawan, etc.) and dental characteristics such as the interincisal angle or the overbite/overjet (Keijirouou, Williams), which are still of the aesthetic profile (Liu, Wendl). The difference in cephalometric measurements used by the authors makes it impossible to exhaustively compare all the skeletal, dento-alveolar, and aesthetic data used for the precise diagnosis of class III malocclusion. The authors of this study recommend the standardization of cephalometric analysis to enable more accurate diagnoses. Another limitation was the follow-up period. Indeed, most of the studies did not have long-term follow-ups of patients following the end of these intercepting treatments. From the selected articles, only Wendl’s retrospective study (20 years of follow-up) and Mandall’s clinical trial (6 years of follow-up) allowed us to draw sufficient conclusions from the data. Indeed, the initial outcomes were successful; however, they differed regarding their long-term stability. Both these studies agreed that SNA, SNB, and ANB values are not maintained over time. We advocate more long-term studies on this theme. It is also important to emphasize that since the initial protocol by Liu et al. in 2005 [35], several variants of the amplitude, frequency, and duration of activation of the circuit breakers have been used in the studies. Unfortunately, there is currently no study on the subject. Indeed, treatment times vary from 4 to 7 weeks, daily activations from 0.4 to 1 mm, and traction forces from 400 to 700 g. It is therefore easy to understand why several studies on the same subject differ in terms of skeletal results. Thus, in this study, the type of Alt-RAMEC protocol used is not specified, as there is a lack of standardization and a lack of agreement and scientific evidence on each of the types [54,55,56,57]. Pure bone-borne orthopedic forces applied with intermaxillary elastics on miniplates were shown to enhance midfacial growth in young maxillary-deficient patients. However, the effectiveness of miniplate treatment could not be tested in this work because no clinical trial with complete cephalometry was found. The results of these treatments seem extremely promising, with point A movements of 4.8 mm [58]. More recently, Cha et al., in 2011, commented on the case of a young patient whose ANB increased from −2.2° to +3.0° at 27 months post-treatment or +5.2° [59]. Faced with the two surgical interventions necessary for this method (installation and removal), other authors, such as Alves de Souza et al. in 2020, have successfully attempted to carry out the same operations using mini-implants. These operations demonstrated that it was possible to perform significant maxillary traction at up to +5° of the ANB angle [60]. The most complete trial to date was carried out in 2016 by Eid et al., where an increase of 2.80° in the SNA angle and a decrease of 0.20° in point B were obtained in a clinical trial. It is essential to continue the investigation of this promising method on a larger scale [61].

## 5. Conclusions

- Alt-RAMEC protocols are recommended for skeletal corrections since they allow better results than traditional maxillary disjunction and traction procedures;
- Moreover, the chin cup has demonstrated limited improvement in patients with early mixed dentitions; therefore, the chin cup is only recommended for treatment in temporary dentition, and a transition to other methods should be made after the eruption of the first permanent molars.

## Figures and Tables

**Table 1:** Cochrane collaboration tool for assessing risk of bias.

Author, Year	Adequate Sequence Generation?	Allocation Concealment?	Participant Blinding?	Blinding of Outcome Assessors?	Incomplete Outcome Data Assessed?	Free of Selective Reporting?	Other Sources of Bias?
Halim, 2023 [30]	Low	Low	Unclear	Low	Low	Unclear	Low
Kajiyama, 2004 [31]	Unclear	Low	Unclear	Low	Low	Unclear	Low
Masucci, 2014 [32]	Low	Low	Unclear	Low	Low	Unclear	Low
Williams, 1997 [33]	Low	Low	Unclear	Low	Low	Unclear	Low
Minase, 2019 [34]	Low	Unclear	Low	Low	Low	Unclear	Low
Liu, 2021 [35]	Low	Low	Unclear	Low	Low	Unclear	Low
Mandall, 2016 [36]	Unclear	Unclear	Unclear	Low	Low	Unclear	Low
Wendl, 2017 [37]	Low	Unclear	Unclear	Low	Low	Unclear	Low

**Table 2:** Qualitative analysis of articles included in the systematic review.

Author/Year	Study Type	Sample and Device (*n*)	Follow-Up Time (Months)	Measurement Procedure	SNA (◦)	SNB (◦)	ANB (◦)
Halim, 2023 [30]	CT	63 RME + Mask	8	Cephalometry	3.32	0.55	2.5
Kajiyama, 2004 [31]	RCT	25 Control	8–24	Cephalometry	1.48	−1.37	2.85
		29 RME + Mask	8–24		0.05	0.82	−0.77
Masucci, 2014 [32]	CT	31 ALT	19	Cephalometry	2.7	1.5	−0.5
		31 RME-FM			−1.5	−0.8	0.5
		21 Control			4.0	2.3	−0.9
Williams, 1997 [33]	CT	28 RME + mask	29	Cephalometry	0.87	0	1.39
Minase, 2019 [34]	RCT	13 RME + RTB	9	Cephalometry	2	1.08	3.7
		13 RME + Mask			1.31	−0.73	2.03
		13 Control			0.66	1.38	−0.58
Liu, 2021 [35]	RCT	13FM	6–8	Cephalometry	1.54	2.25	3.75
		13 RME-FM	6–8	Cephalometry	2.42	−1.17	3.92
		13 ALT	6–8	Cephalometry	3.30	−1.03	3.47
Mandall, 2016 [36]	RCT	35 FM	72	Cephalometry	1.10	−1.5	2.6
		38 Control	72		1.30	0.8	−0.50
Wendl, 2017 [37]	CT	23 RME+ FM	180	Cephalometry	1.40	−0.7	1.70
		38 Chin	180–240	Cephalometry	0.40	−0.60	0.90

RCT, randomized controlled trial; CT, clinical trial; FM, facial mask; RME, rapid maxillary expansion; ALT, Alt-RAMEC procedure; RTB, reverse twin block; Chin, chin cup.

## Data Availability

Information is available upon request in accordance with relevant restrictions (e.g., privacy or ethics).

## References

[1] Jawad Z., Bates C., Hodge T. (2015). Who needs orthodontic treatment? Who gets it? And who wants it?. Br. Dent. J..

[2] John H. (1778). Natural History of the Human Teeth.

[3] Amat P. (2013). Traitement précoce des malocclusions de classe III : Les faits. Orthod. Fr..

[4] Zere E., Chaudhari P.K., Sharan J., Dhingra K., Tiwari N. (2018). Developing Class III malocclusions: Challenges and solutions. Clin. Cosmet. Investig. Dent..

[5] Londono J., Ghasemi S., Moghaddasi N., Baninajarian H., Fahimipour A., Hashemi S., Fathi A., Dashti M. (2023). Prevalence of malocclusion in Turkish children and adolescents: A systematic review and meta-analysis. Clin. Exp. Dent. Res..

[6] Vesse M. (2007). Classes III squeletiques. Elsevier Masson SAS Paris Odontol Dentofaciale.

[7] Kassas A. (2022). Critères épidémiologiques de la dysmorphose de classe III chez les patients âgés de 6 à 14 ans. Rev. Médicale L’hmruo.

[8] Fakharian M., Bardideh E., Abtahi M. (2019). Skeletal Class III malocclusion treatment using mandibular and maxillary skeletal anchorage and intermaxillary elastics: A case report. Dent. Press J. Orthod..

[9] Jamilian A., Cannavale R., Piancino M.G., Eslami S., Perillo L. (2016). Methodological quality and outcome of systematic reviews reporting on orthopaedic treatment for class III malocclusion: Overview of systematic reviews. J. Orthod..

[10] Staudt C.B., Kiliaridis S. (2009). Different skeletal types underlying Class III malocclusion in a random population. Am. J. Orthod. Dentofac. Orthop..

[11] Azamian Z., Shirban F. (2016). Treatment Options for Class III Malocclusion in Growing Patients with Emphasis on Maxillary Protraction. Scientifica.

[12] Rutili V., Quiroga Souki B., Nieri M., Farnese Morais Carlos A.L., Pavoni C., Cozza P., McNamara J.A., Giuntini V., Franchi L. (2023). Long-Term Assessment of Treatment Timing for Rapid Maxillary Expansion and Facemask Therapy Followed by Fixed Appliances: A Multicenter Retro-Prospective Study. J. Clin. Med..

[13] Jaradat M. (2018). An Overview of Class III Malocclusion (Prevalence, Etiology and Management). J. Adv. Med. Med. Res..

[14] Achalli S., Nayak U.K., Murali P.S., Shashidhar K., Kamath V. (2023). Comparative evaluation of dermatoglyphic patterns between skeletal class I and skeletal class III malocclusion. F1000Research.

[15] Peng H., Liu W., Yang L., Yan P., Zhong W., Gao X., Song J. (2024). Craniocervical posture in patients with skeletal malocclusion and its correlation with craniofacial morphology during different growth periods. Sci. Rep..

[16] Rodríguez-Olivos L.H.G., Chacón-Uscamaita P.R., Quinto-Argote A.G., Pumahualcca G., Pérez-Vargas L.F. (2022). Deleterious oral habits related to vertical, transverse and sagittal dental malocclusion in pediatric patients. BMC Oral Health.

[17] Lin L., Zhao T., Qin D., Hua F., He H. (2022). The impact of mouth breathing on dentofacial development: A concise review. Front. Public Health.

[18] Santos Barrera M., Ribas-Perez D., Caleza Jimenez C., Cortes Lillo O., Mendoza-Mendoza A. (2024). Oral Habits in Childhood and Occlusal Pathologies: A Cohort Study. Clin. Pract..

[19] Seifi M., Hamedi R., Khavandegar Z. (2015). The Effect of Thyroid Hormone, Prostaglandin E2, and Calcium Gluconate on Orthodontic Tooth Movement and Root Resorption in Rats. J. Dent..

[20] Li Z., Hung K.F., Ai Q.Y.H., Gu M., Su Y.-X., Shan Z. (2024). Radiographic Imaging for the Diagnosis and Treatment of Patients with Skeletal Class III Malocclusion. Diagnostics.

[21] Demircan G.S., Kılıç B., Önal-Süzek T. (2020). Early diagnosis and prediction of skeletal class III malocclusion from profile photos using artificial intelligence. IFMBE Proc..

[22] Abdelnaby Y.L., Nassar E.A. (2010). Chin cup effects using two different force magnitudes in the management of Class III malocclusions. Angle Orthod..

[23] Gazzani F., Pavoni C., Giancotti A., Cozza P., Lione R. (2018). Facemask performance during maxillary protraction: A finite element analysis (FEA) evaluation of load and stress distribution on Delaire facemask. Prog. Orthod..

[24] Lee N.K., Kim S.H., Park J.H., Son D.W., Choi T.H. (2023). Comparison of treatment effects between two types of facemasks in early Class III patients. Clin. Exp. Dent. Res..

[25] Liou E.J., Tsai W.C. (2005). A new protocol for maxillary protraction in cleft patients: Repetitive weekly protocol of alternate rapid maxillary expansions and constrictions. Cleft Palate-Craniofacial J..

[26] Almeida R.R., Alessio L.E., Almeida-Pedrin R.R., Almeida M.R., Pinzan A., Vieira L.S. (2015). Management of the Class III malocclusion treated with maxillary expansion, facemask therapy and corrective orthodontic. A 15-year follow-up. J. Appl. Oral Sci..

[27] De Clerck H.J., Cornelis M.A., Cevidanes L.H., Heymann G.C., Tulloch C.J. (2009). Orthopedic traction of the maxilla with miniplates: A new perspective for treatment of midface deficiency. J. Oral Maxillofac. Surg..

[28] Page M.J., McKenzie J.E., Bossuyt P.M., Boutron I., Hoffmann T.C., Mulrow C.D., Shamseer L., Tetzlaff J.M., Akl E.A., Brennan S.E. (2021). The PRISMA 2020 statement: An updated guideline for reporting systematic reviews. BMJ.

[29] Higgins J.P.T., Green S. (2011). Cochrane Handbook for Systematic Reviews of Interventions Version 5.1.0.

[30] Halim H., Halim I.A. (2023). Profile Changes in Class III Malocclusion using Protraction Facemask in Indonesian Young Patients (Cephalometric Study). Open Dent. J..

[31] (2004). Kajiyama K, Murakami T, Suzuki A. Comparison of orthodontic and orthopedic effects of a modified maxillary protractor between deciduous and early mixed dentitions. Am. J. Orthod. Dentofac. Orthop..

[32] Masucci C., Franchi L., Giuntini V., Defraia E. (2014). Short-term effects of a modified Alt-RAMEC protocol for early treatment of Class III malocclusion: A controlled study. Orthod. Craniofac. Res..

[33] Williams M.D., Sarver D.M., Sadowsky P.L., Bradley E. (1997). Combined rapid maxillary expansion and protraction facemask in the treatment of Class III malocclusions in growing children: A prospective long-term study. Semin. Orthod..

[34] Minase R.A., Bhad W.A., Doshi U.H. (2019). Effectiveness of reverse twin block with lip pads-RME and face mask with RME in the early treatment of class III malocclusion. Prog. Orthod..

[35] Liu Y., Hou R., Jin H., Zhang X., Wu Z., Li Z., Guo J. (2021). Relative effectiveness of facemask therapy with alternate maxillary expansion and constriction in the early treatment of Class III malocclusion. Am. J. Orthod. Dentofac. Orthop..

[36] Mandall N., Cousley R., DiBiase A., Dyer F., Littlewood S., Mattick R., Nute S.J., Doherty B., Stivaros N., McDowall R. (2016). Early class III protraction facemask treatment reduces the need for orthognathic surgery: A multi-centre, two-arm parallel randomized, controlled trial. J. Orthod..

[37] Wendl B., Stampfl M., Muchitsch A.P., Droschl H., Winsauer H., Walter A., Wendl M., Wendl T. (2017). Long-term skeletal and dental effects of facemask versus chincup treatment in Class III patients: A retrospective study. J. Orofac. Orthop..

[38] Hagg U., Tse A., Bandeus M., Rabie A.B. (2003). Long-term follow-up of early treatment with reverse headgear. Eur. J. Orthod..

[39] McNamara J.A., Graber T.M., Vanarsdall R.L. (1994). Mixed dentition treatment. Orthodontics-Current Principles and Techniques.

[40] Hickam J.H. (1991). Maxillary protraction therapy: Diagnosis and treatment. J. Clin. Orthod..

[41] Kim J.H., Viana M.A.G., Graber T.M., Omerza F.F., BeGole E.A. (1999). The effectiveness of protraction fece mask therapy: A meta-analysis. Am. J. Orthod. Dentofac. Orthop..

[42] Bacetti T., McGill J.S., Franchi L., McNamara J.A., Tollaro I. (1998). Skeletal effects of early tretment of Class III malocclusions with maxillary expansion and face-mask therapy. Am. J. Orthod. Dentofac. Orthop..

[43] Sung S.J., Baik H.S. (1998). Assessment of skeletal and dental changes by maxillary protraction. Am. J. Orthod. Dentofac. Orthop..

[44] Isci D., Turk T., Elekdag-Turk S. (2010). Activation-deactivation rapid palatal expansion and reverse headgear in Class III cases. Eur. J. Orthod..

[45] Zhao T., Hua F., He H. (2020). Alternate Rapid Maxillary Expansion and Constriction (Alt-RAMEC) May Be More Effective Than Rapid Maxillary Expansion Alone for Protraction Facial Mask Treatment. J. Evid. Based Dent. Pract..

[46] Petrovic A.G., Stutzmann J.J., Oudet C.L., McNamara J.A. (1975). Control process in the postnatal growth of the condylar cartilage of the mandible. Determinants of Mandibular Form and Growth.

[47] Chang H.P., Lin H.C., Liu P.H., Chang C.H. (2005). Geometric morphometric assessment of treatment effects of maxillary protraction combined with chin cup appliance on the maxillofacial complex. J. Oral Rehabil..

[48] Ngan P., Bishara E.S. (2001). Treatment of Class III malocclusion in the primary and mixed dentitions. Textbook of Orthodontics.

[49] Proffit W.R., Fields H.W. (2000). Contemporary Orthodontics.

[50] McNamara J.A., Graber T.M., Vanarsdall R.L., Vig K.W.L. (2005). Treatment of patients in the mixed dentition. Orthodontics: Current Principles and Techniques.

[51] Sugawara J., Nanda R. (2005). Clinical practice guidelines for developing Class III malocclusion. Biomechanics and Esthetic Strategies in Clinical Orthodontics.

[52] Husson A.H., Burhan A.S., Hajeer M.Y., Nawaya F.R. (2023). Evaluation of the dimensional changes in the mandible, Condyles, and the temporomandibular joint following skeletal class III treatment with Chin Cup and bonded maxillary bite block using low-dose computed tomography: A single-center, Randomized Controlled Trial. F1000Research.

[53] Barrett A.A., Baccetti T., McNamara J.A. (2010). Treatment effects of the light-force chincup. Am. J. Orthod. Dentofac. Orthop..

[54] Buyukcavus M.H. (2019). Alternate Rapid Maxillary Expansion and Constriction (Alt-RAMEC) protocol: A Comprehensive Literature Review. Turk. J. Orthod..

[55] Pithon M.M., Santos N.d.L., dos Santos C.R.B., Baião F.C.S., Pinheiro M.C.R., Neto M.M., Souza I.A., de Paula R.P. (2016). Is alternate rapid maxillary expansion and constriction an effective protocol in the treatment of class III malocclusion? A systematic review. Dent. Press J. Orthod..

[56] Al-Mozany S., Tarraf N., Dalci O., Gonzales C., Darendeliler M.A. (2011). Treatment of Class III Malocclusions Using Temporary Anchorage Devices (TADs) and Intermaxillary Class III Elastics in the Growing Patient. Ph.D. Thesis.

[57] Kaya D., Kocadereli I., Kan B., Tasar F. (2011). Effects of facemask treatment anchored with miniplates after alternate rapid maxillary expansions and constrictions; a pilot study. Angle Orthod..

[58] Kircelli B.H., Pektas Z.O. (2008). Midfacial protraction with skeletally anchored face mask therapy: A novel approach and preliminary results. Am. J. Orthod. Dentofac. Orthop..

[59] Cha B.-K., Choi D.-S., Ngan P., Jost-Brinkmann P.-G., Kim S.-M., Jang I.-S. (2011). Maxillary protraction with miniplates providing skeletal anchorage in a growing class III patient. Am. J. Orthod. Dentofac. Orthop..

[60] Souza R.A., Dourado G.B., Farias I.M., Pithon M.M., Neto J.R., de Paiva J.B. (2020). Miniscrews as an alternative for orthopedic traction of the maxilla: A case report. APOS Trends Orthod..

[61] Eid O.M., Ramadan A.A.-F., Nadim M.A., Hamed T.A.-B. (2016). Maxillary protraction using orthodontic miniplates in correction of class III malocclusion during growth. J. World Fed. Orthod..

